# Mesial temporal sclerosis and epilepsy: a narrative review

**DOI:** 10.1186/s42494-024-00172-5

**Published:** 2024-09-15

**Authors:** Daniel Villamizar-Torres, Andrea Carolina Cepeda Trillos, Alejandro Vargas-Moreno

**Affiliations:** 1 Member of the neurosurgery research group, Pontificia Universidad Javeriana, Bogotá, 110231 Colombia; 2 Present Address: Radiology deparment, Hospital Universitario San Ignacio, Bogotá, 110231 Colombia; 3 Present Address: Member of the epilepsy research group, Pontificia Universidad Javeriana, Bogotá, 110231 Colombia; 4 Neurosurgery department, Hospital Universitario San Ignacio, Bogotá, 110231 Colombia

**Keywords:** Hippocampal sclerosis, Epilepsy, Temporal lobe, Hippocampus

## Abstract

Mesial temporal sclerosis (MTS) stands out as a prevalent etiology of medically intractable temporal lobe epilepsy. Understanding the pathological alterations, clinical manifestations and risk factors of MTS is crucial for the recognition and suspicion of this condition. In this paper, we provide a comprehensive narrative review on the pathophysiology, clinical manifestations, and treatment options for MTS. By doing so, we aim to provide an up-to-date understanding of this condition.

## Background

 The hippocampus has long been recognized as a structure that serves as both the primary site of epileptic seizures and as a region particularly vulnerable to secondary damage resulting from seizures or other brain injuries such as mesial temporal sclerosis (MTS). The characteristic gliosis, neuronal loss, and reorganization of the dentate gyrus and hippocampal circuits in MTS make it the second most common cause of temporal lobe epilepsy (TLE) in children. MTS is also the primary indication for surgical intervention in patients with epilepsy [1–3].

A comprehensive search was made in the PubMed, Embase, and Google Scholar databases, using keywords such as “Mesial Temporal Sclerosis”, “Hippocampal Sclerosis”, and “Temporal Lobe Epilepsy”. The articles were carefully chosen based on their quality, suitability, and relevance to the topics of pathophysiology, epidemiology, clinical presentation, diagnosis, and management. References from eligible articles were also reviewed to include suitable articles. In total, 106 articles published from 2004 to 2024 were included in this review, ensuring a thorough and comprehensive analysis.

## Main text

### Definition

The term MTS was introduced by Falconer et al. to describe neuronal loss and gliosis in the anterior temporal lobes, as observed in surgical samples from a cohort of 100 patients receiving surgery for intractable TLE. These pathological changes represented the most common pathology identified within the cohort [2]. MTS is characterized by significant loss of neurons in the hilus of the dentate gyrus and pyramidal neurons in the Ammon’s horn of the hippocampus, particularly in the CA1 and CA3 regions [3].

### Historical understanding of MTS and its associations with epilepsy

The association between neuronal loss in these specific areas and epilepsy was acknowledged over a century ago. In 1825, Bouchet and Cazauvielh initially reported macroscopic structural alterations in the mesial temporal region in 14 out of 18 autopsies conducted on patients who experienced seizures associated with “mental alienation” [4].

Subsequently, in 1880, Sommer made significant contributions by describing the histopathological changes in MTS. He identified the distinctive pattern of neuronal loss that is characteristic of MTS, with a particular emphasis on prominant neuronal loss in the CA1 region, which became known as “Sommer’s sector” [5].

However, these pathological findings were not specific and were not yet linked to any specific type of epilepsy. It wasn’t until 1936 when Stauder made the correlation between sclerosis of Ammon’s horns and TLE [6], establishing a crucial connection between the observed pathological changes and the specific form of epilepsy.

### Epidemiology

Epilepsy has a prevalence ranging from 0.5 to 1% in the pediatric population [7], with an annual incidence of 33 to 82 cases per 100,000 children [8]. Among children with epilepsy, up to 20% may have TLE, making it the most common form of focal epilepsy. Specifically, mesial temporal epilepsy, which typically manifests between the ages 6 and 20, can occur at any age [9, 10].

The causes of drug-resistant epilepsy in children can be classified into three main groups. MTS ranks as the second most common cause, accounting for approximately 23–29% of cases, only next to malformation of cortical development and low-grade epilepsy-associated tumors, which are observed in approximately 40% of cases [1, 11, 12].

It is important to note that approximately one-third of epilepsy patients are resistant to drug treatment [13], often due to the underlying brain lesions. Among children, around 15.5% would qualify as candidates for surgical resection [14].

Therefore, it is crucial to recognize these pathologies, especially MTS, in order to provide a more effective approach for drug-resistant epilepsy in the pediatric population.

### Pathophysiological relationship

**Fig. 1:**
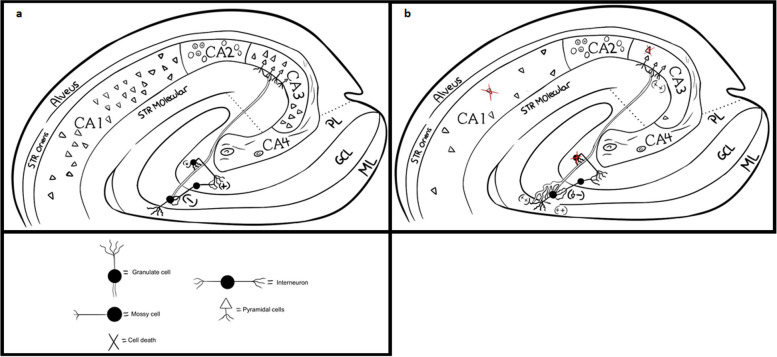
**a** Illustration of normal synaptic connections between dentate - hippocampal circuits. **b** Illustration of pathological changes in MTS, including mossy fibers sprouting, mossy cell loss and loss of pyramidal cells in CA1 and CA3. (-) = Inhibitory, (+) = excitatory. Abbreviations: GCL: Granule cell layer, ML: Molecular layer, Str: Stratum, PL: Polymorphic layer

It has been recognized that the spread of seizures from remote areas of the cerebral cortex to the hippocampus is sufficient to disrupt synaptic connections in the mossy fibers [15]. Severe damage, such as that caused by status epilepticus, can even result in macroscopic lesions in the hilus of the dentate gyrus and hippocampal subregions [16]. Consequently, the mossy fibers progressively ramify, leading to alterations in synaptic connectivity and the reorganization of circuits within the inner molecular layer of the dentate gyrus. This process leads to the formation of recurrent excitatory connections that contribute to an increased susceptibility to seizures (Fig. 1) [17].

In addition to neuronal branching, seizures are also associated with apoptosis and neuronal death in regions such as CA1, CA3, the subiculum, and the dentate gyrus [18]. Olney conducted extensive studies on the effects of kainate-induced seizures in the brain. Kainate, an analogue of glutamate, causes significant damage in the hippocampus when injected in the brain. He proposed that the brain damage associated with seizures is mediated through a process of endogenous excitotoxicity caused by sustained release of glutamate at synaptic terminals [19].

Studies have demonstrated that mossy cells are particularly vulnerable to excitotoxicity [20]. Electrophysiological studies have revealed that these mossy cells play a crucial role in innervating GABAergic interneurons, which regulate the activity of granule cells [21]. The selective loss of mossy neurons leads to a disruption of lateral inhibition on granule cells. As a result, the granule cells become hyperexcitable and disinhibited in the presence of orthodromic stimulation [22].

These findings support the notion that MTS is both a cause and a consequence of seizures. Prolonged seizure activity induces an injury that triggers synaptic reorganization, leading to hyperexcitability of neuronal circuits and increased susceptibility to epileptic seizures. This forms a vicious cycle, resulting in refractory epilepsy [23, 24].

Although MTS has long been considered a unilateral pathology, there is evidence supporting the bilateral involvement. Babb and Brown found that 80% of their studied cohort had asymmetric bilateral damage, 10% had symmetric involvement of both hippocampi, and only 10% had unilateral involvement [25].

The existence of dual pathology in the temporal lobe is common. The incidence of MTS and an extrahippocampal lesion ranges from 8 to 22%. MTS is frequently associated with other conditions such as temporal hamartomas, cortical developmental malformations (e.g., focal cortical dysplasias), vascular malformations, among others [26–28]. In children, the incidence of dual pathology is even higher, ranging from 31 to 79%, with cortical dysplasias and low-grade tumors being the most common lesions in association with MTS [29–31].

### Risk factors

Cavanagh and Meyer reported a higher frequency of MTS in adults who experienced early onset seizures before the age of 4, with a stronger association in those who had experienced status epilepticus [32]. Building upon this, Babb and Brown hypothesized that early seizures could lead to neuronal damage and reactive gliosis in the hippocampus [25]. This sparked a debate in the medical literature regarding the relationship between MTS and epilepsy. Two main hypotheses were proposed. One suggested that early febrile seizures in childhood can cause damage to the hippocampus, thus being the cause of MTS, while the other proposed that a predisposed hippocampus in a child suffering prolonged febrile seizures was the fundamental cause [23].

Subsequent studies, including the FEBSTAT12 study, provided further evidence of acute hippocampal injury following febrile seizures, as demonstrated by T2 hyperintensity in Sommer’s sector and impaired hippocampal growth in affected patients [30]. In addition, studies have revealed increased cytokines such as IL-1β, IL-18, CCL2, CCL3, and CCL4 in epileptic patients, highlighting inflammation as an important modulator in epileptogenesis [31]. Moreover, studies have elucidated autoimmune encephalitis, specially voltage-gated potassium channel complex encephalitis, as a possible generator of MTS [33].

Furthermore, there is extensive literature on initial precipitating lesions (IPLs), which are defined as significant clinical events (cerebral diseases or injuries associated with prolonged alterations in consciousness and/or major cognitive impairments) that occur prior to the onset of TLE [34]. These IPLs play a crucial role in initiating the vicious cycle leading to the development of MTS.

Most of the data regarding IPL have been obtained from retrospective cohorts of patients with MTS who underwent surgical management. In the study by Blümcke et al., among 171 patients with MTS, 39.2% had a clear history of IPL, with 56.7% having a history of complex febrile seizures, 22.4% with a history of encephalitis, 10.4% with head trauma, and 6% with birth trauma or intracranial bleeding [35]. It has been reported that patients who experienced an initial symptomatic seizure secondary to meningitis, trauma, prolonged seizures, or febrile seizures are at an increased risk of developing MTS [34].

In addition to the type of noxious event, the age of onset is also an important factor, as IPLs that occur in earlier stages of childhood are associated with greater neuronal loss [36]. Sagar et al. grouped 32 temporal lobectomy samples from patients with MTS based on the age of first seizure occurrance (before 3 years vs. after 4 years). They found a significantly lower neuronal count in the CA1 zone, dentate gyrus, and folium in the group of < 3 years, which also exhibited longer seizure durations. These findings highlight the strong association between MTS and the history of seizures in early childhood [37].

To establish a classification system that influences clinical management and outcomes, Mathern et al. classified IPL into five types (Table 1). According to this classification, groups 1 and 2 show a higher prevalence of hippocampal atrophy and mossy fiber sprouting compared to the other groups. Furthermore, patients with non-convulsive IPL had a worse response to surgical management, and those with idiopathic MTS experienced even poorer outcomes [34].

**Table 1:** Classification of initial precipitating lesions (IPLs) described by Mathern et al. [34]

IPL type	Presentation
Nonseizure IPL	Clinically significant events that occurred under 5 years of age, not associated with seizures.
Prolonged seizure IPL	Clinically significant events that occurred under 5 years of age, associated with a motor seizure secondary to status epilepticus or a complex febrile seizure.
Non-prolonged seizure IPL	Multiple non-significant seizures with a brief loss of consciousness, not suggestive of status epilepticus
Late IPL	All IPL that occurred over 5 years of age
Idiopathic MTS	No history of clinically significant events during life

The role of genetics in the development of MTS has gained attention. It is now recognized that genetic predispositions may be necessary for the development of hippocampal sclerosis as a consequence of a noxious event. Polymorphisms in the *SCN1A* gene, which codes for α-subunit of the neuronal voltage-gated sodium ion channel type 1, have been associated with an increased risk of febrile seizures and MTS [38]. This suggests that the development of MTS involves complex interactions between genetic factors and noxious events during childhood [39]. In addition, some studies have proposed that epigenetic-mediated gene output may be related to epileptogenesis in MTS. These outputs include alterations in ion channels, metabolism and neurotransmitters, inflammation, altered signaling, and oxidative stress, with mechanisms involving hyper- or hypo-methylation [40].

Furthermore, a correlation between MTS and hematological malignancies, with or without stem cell transplantation, has been established. The use of intrathecal chemotherapy in these cases has been identified as a potential precipitating factor for MTS [41]. This highlights the role of treatment-related complications in the development of MTS.

### Classification

MTS is not a single disease entity, and its classification requires consideration of multiple variables. Recent classifications have emphasized not only the percentages of neuronal loss but also the patterns of neuronal loss and gliosis [42].

Building upon the findings of Blümcke et al. [43], the International League Against Epilepsy (ILAE) released a classification system in 2013 for MTS, which categorizes MTS into four types, with a correlation to the age of IPL (Table 2) [43, 44]. This classification system aims to provide a more comprehensive understanding of MTS and its relationship to IPL based on the age of presentation.

**Table 2:** 2013 International League against Epilepsy classification of MTS and the average age of initial precipitating lesion (IPL) occurrence [43, 44]

MTS classification	Age of IPL	Description
Type 1 (classic): 60–80% of cases	< 3 years	CA1-predominant severe loss of pyramidal neurons (PNs); also significant PN loss in CA2, CA3 and CA4
Type 2 5–10% of cases	6 years	CA1-predominant PN loss; slight cell loss in the other sectors
Type 3 (folium terminal sclerosis)	> 13 years	Predominant PN loss in CA4 and the dentate gyrus, associated with moderate loss of PN in the other sectors.
Type 4 (No- hippocampal sclerosis)	> 16 years	No evidence of hippocampal sclerosis; only focal gliosis

### Clinical manifestations

MTS were initially described by Gibbs in 1937 as an electroclinical syndrome known as “psychomotor seizures” [45]. Seizures associated with MTS are typically focal seizures, often characterized by impaired alertness. They typically last about 1–2 min and rarely progress to secondary generalized tonic-clonic seizures. These seizures are often preceded by auras in the form of epigastric discomfort, fear, anxiety, and autonomic symptoms. Patients may also experience depersonalization, déjà vu, dysphoric or euphoric feelings. When awareness is impaired, behavioral arrest and automatisms may occur, followed by a postictal period [46].

The electroencephalogram (EEG) of MTS may include intermittent periods of slowing in the anterior temporal electrode contacts with rhythmic delta or theta activity, as well as spikes or sharp waves over the anterior temporal regions, which are more commonly seen during wakefulness and early stages of sleep. The seizure activity can begin in the anterior temporal or even sphenoidal electrodes and then spread to the lateral temporal, insular, and frontal lobes. There can be no electrical abnormalities during psychogenic seizures [46, 47].

**Fig. 2:**
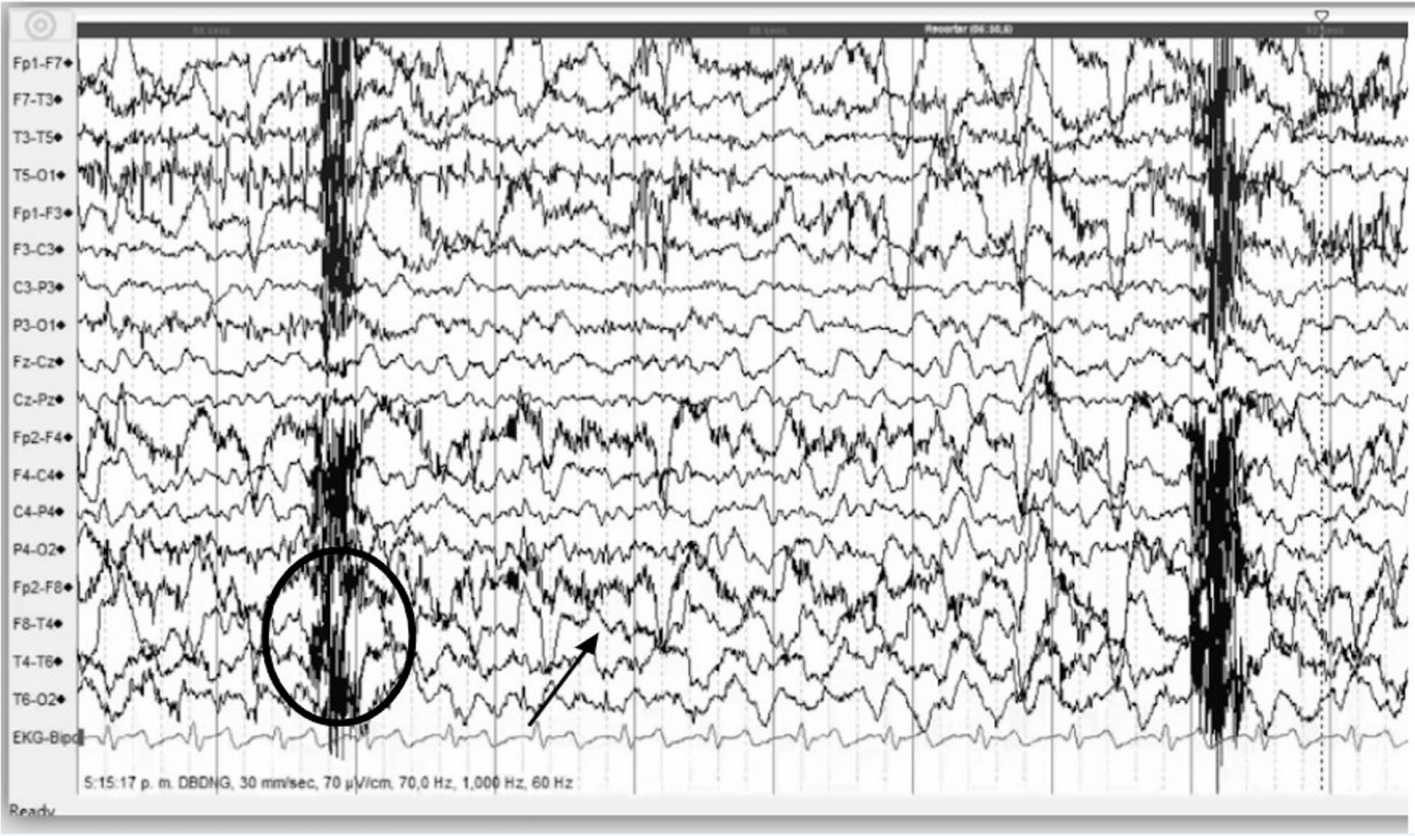
EEG recording in a patient with diagnosis of mesial temporal sclerosis during a seizure at the awake state. The EEG showed a right temporal electrical onset with ipsilateral central-frontal irradiation at a speed of 2–3 cycles per second (cps), with medium- to high-amplitude spikes (arrow). Electromyogram artifacts are observed in temporal regions (circled area)

An example of EEG recording in a patient diagnosed with MTS during non-motor focal onset seizures with impaired alertness and orolingual automatisms is shown in Fig. 2.

### Brain imaging findings

In patients with mesial temporal lobe epilepsy (MTLE), the presence of hippocampal sclerosis (HS) on preoperative magnetic resonance imaging (MRI) has been associated with adequate seizure control as well as favorable postoperative outcomes [48–50]. MRI is considered the gold standard for HS diagnosis, ruling out alternative pathologies that may cause MTLE [51].

**Fig. 3:**
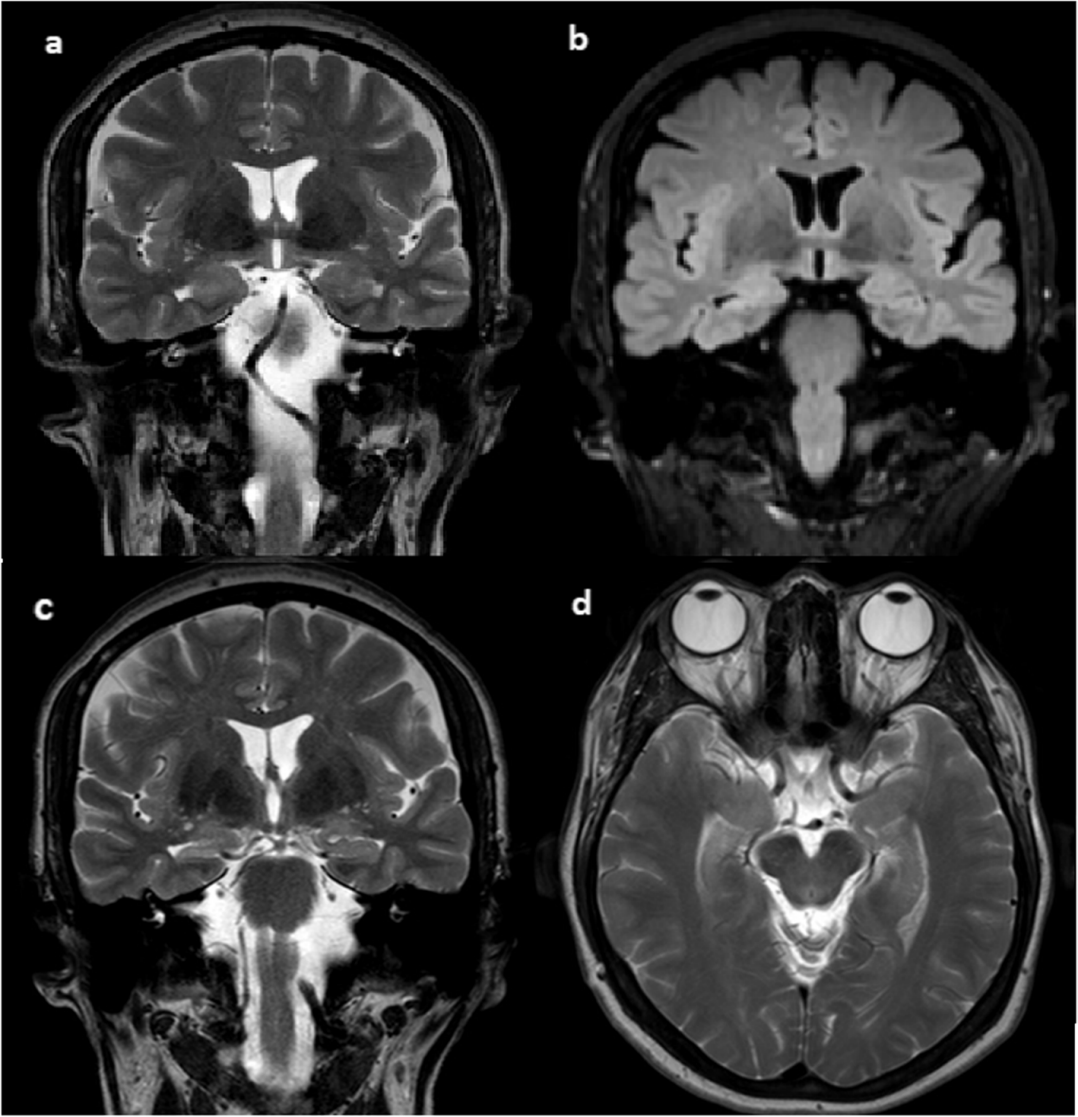
Coronal magnetic resonance images showing marked asymmetry of the hippocampal heads (**a**, **b**), with atrophy in the right side. Additionally, there is an increased signal intensity in both T2WI (**a**) and FLAIR WI (**b**). (**c**) Coronal T2WI image showing a normal left hippocampus and a flattened, atrophic right hippocampal body with dilatation of the adjacent ipsilateral temporal horn, also seen in axial T2WI (**d**). The features are consistent with mesial temporal sclerosis

For qualitative assessment of temporal lobe abnormalities on standard MRI, an epilepsy protocol is typically employed, which involves acquiring thin coronal slices perpendicular to the long axis of the hippocampus. This imaging approach allows for better visualization of the relevant structures [48]. The most common qualitative MRI findings in HS (Fig. 3) include:

1. Hippocampal atrophy: This finding is present in approximately 90–95% of HS cases. Hippocampal atrophy is established by comparing the circumference of the hippocampus on each side and assessing the shape of the structure on coronal section imaging. In MTS, the hippocampal body has a flattened structure instead of a normal oval shape [48].
2. Increased signal intensity on T2-weighted imaging (WI): This finding is observed in approximately 80–85% of HS cases and is thought to reflect glial changes in the cytoarchitecture of the hippocampus and dentate gyrus [52].
3. Disturbed internal architecture: This finding is observed in approximately 60–95% of HS cases and is characterized by reduced demarcation between gray matter and white matter as well as loss of the interdigitations of the hippocampus. These changes contribute to the overall atrophy and structural alterations seen in HS [53–61].
4. Decreased T1-weighted signal: A decrease in T1-weighted signal intensity is observed in approximately 10–95% of HS cases. This finding is indicative of tissue changes and can further support the diagnosis [53–61].

While less common, extra-hippocampal structures within the limbic system may also be involved in HS, including ipsilateral atrophy of the fornix, the mammillary bodies, the amygdala, the anterior thalamic nuclei, and the cingulate gyrus. Extralimbic findings such as increased signals in the anterior temporal lobe cortex and cerebral hemiatrophy have also been described [62].

It is important to note that the MRI findings in HS should be interpreted cautiously and in conjunction with the clinical context and electroclinical findings. False positives may exist, and MRI evidence of HS can occur in individuals who have never experienced seizures [58, 59]. Therefore, a comprehensive evaluation incorporating all available clinical and imaging information is necessary for accurate diagnosis and treatment planning.

Multiple case-control studies have been conducted to evaluate the frequency of mesial temporal abnormalities on brain MRI in healthy individuals compared to patients with TLE. FLAIR hyperintensity and unilateral temporal horn dilatation may be observed in up to one-third of normal controls [63]. The presence of hippocampal atrophy in conjunction with hyperintensity is the strongest and most reliable indicator of epilepsy [60].

Qualitative evaluation of MTS on MRI has limitations in cases of mild sclerosis or bilateral atrophy according to the sensitivity and predictive values [60]. Objective quantitative assessment methods, such as assessment by hippocampal volumetry and T2 relaxometry, can improve the detection of subtle atrophy and signal changes that may not be visually identifiable [62]. These quantitative measures can also be used for evaluating contralateral hippocampal integrity, as bilateral hippocampal abnormalities are associated with poor seizure control following anterior temporal lobe resection and an increased risk of memory impairment [64].

Volumetric quantification can be performed using automated methods or with manual measurements [65]. Studies have demonstrated that the hippocampal volumetric index and hippocampal asymmetry index yield better sensitivity and specificity compared to qualitative assessment [65, 66]. Kuzniecky et al. have found a statistically significant difference in the sensitivity between quantitative methods and qualitative visual identification of atrophy [49].

In some patients with chronic TLE, approximately 15–30% of individuals with HS may not exhibit MTS on volumetric assessment of the hippocampus. These patients are referred to as MRI-negative TLE [67]. In such cases, invasive studies like intracranial EEG recording may be necessary to identify the epileptogenic zone, but this approach has risks of complications such as intracranial infection and hemorrhage [68]. To avoid the use of invasive diagnostic procedures, various functional studies have been extensively investigated.

Proton-magnetic resonance spectroscopy (PMRS) is one of the most commonly used modalities for functional evaluation. It assesses neuronal integrity by measuring N-acetylaspartate (NAA), a neuronal marker sensitive to neuronal loss or dysfunction. PMRS compares the concentrations of NAA with choline (Cho) or creatine (Cr). In patients with MTS, there is a decreased concentration of NAA and increased concentrations of Cho and Cr due to hippocampal gliosis and neuronal loss [82]. The main findings from PMRS include ipsilateral hippocampal decrease of NAA, decreased NAA/(Cho + Cr) ratio, decreased NAA/Cr ratio, and extra-hippocampal NAA decreases in temporal lobe white matter, insula, thalamus, frontal lobe, and even the contralateral hippocampus [69]. In a study by Fountas et al., PMRS detected MTS with a sensitivity of 100%, a specificity of 80%, a positive predictive value (PPV) of 87%, and a negative predictive value (NPV) of 100% [70]. However, the use of PMRS for preoperative evaluation of patients with MTS, particularly for selecting the most appropriate surgical strategy, is not yet clear. Only one meta-analysis by Willmann et al. demonstrated that ipsilateral abnormalities in PMRS are associated with good outcomes after surgery, but further research is needed in this field [71].

In the late 20th century, ^18^F-fluorodeoxyglucose positron emission tomography (FDG-PET) emerged as an important tool for localizing seizure onset zones in TLE [72]. FDG-PET measures interictal glucose metabolism in the cerebral parenchyma. In MTS, a characteristic finding is the diffuse regional hypometabolism in both mesial and lateral temporal structures [73]. Studies comparing FDG-PET and PMRS have shown similar abilities to accurately diagnose MTS [74, 75]. Park et al. demonstrated a sensitivity of 85% by FDG-PET for lateralization of the epileptogenic focus, which is consistent with the reported sensitivity of 85% for MRS; however, FDG-PET showed a higher proportion of false positives [75]. A meta-analysis by Willmann et al. suggested that ipsilateral hypometabolism on FDG-PET in presurgical evaluation may be indicative of a good postoperative outcome, but further research is needed to validate these results [76].

In children, MRI-assissted diagnosis has not been extensively studied. Kasasbeh et al. evaluated the value of MRI in preoperative surgical evaluation and reported a PPV of 55–67% and a NPV of 79–87%. Similar to adults, the predictive value was highest for increased hippocampal signal and reduced hippocampal size, although the prevalence of MRI findings indicative of MTS was lower in children [77]. The hippocampal size reduction may be more subtle in children compared to that in adults due to the progression and maturation of MTS lesions, making the diagnosis more challenging in children.PMRS has shown promise in adequately diagnosing lateralization of the seizure focus and detecting bilateral abnormalities in children [78, 79].

Overall, both FDG-PET and PMRS have demonstrated value in the preoperative evaluation of MTS, providing additional information to aid in the localization and characterization of epileptogenic zones. Further research is needed to improve their utility and verify their roles in guiding treatment decisions in both adult and pediatric populations.

### Treatment

Epilepsy secondary to MTS is associated with a poor prognosis, with limited potential for seizure control through anticonvulsant medications [80]. A study examining 550 patients with focal epilepsy secondary to various etiologies treated with anticonvulsant medications showed that patients with MTS had a significantly higher risk of poor seizure control compared to other etiologies such as arteriovenous malformations, cerebral infarcts, cortical gliosis, and brain tumors. Additionally, they had a higher risk of requiring multiple anticonvulsant medications [81].

The goal of antiepileptic management is to achieve seizure control. A randomized controlled study comparing surgical resection with anticonvulsant medication in patients with mesial temporal epilepsy showed that the proportions of patients achieving seizure freedom at one year were 42% in the surgery group and 8% in the medication group. Additionally, the surgical group had a reduced risk of sudden unexpected death in epilepsy [82].

Surgery for MTLE involves resection of the hippocampus, the amygdala, and the parahippocampal gyrus, although the extent of resection is not standardized [83]. There are two surgical options: anterior temporal lobectomy (ATL) and selective amygdalohippocampectomy (SAH). ATL involves resection of the anterior temporal cortex and posterior mesial structures [84]. SAH aims to precisely remove the tissues directly responsible for epileptogenesis without affecting the surrounding neocortex that may be involved in language and cognition, thus preserving neuropsychological outcomes [85].

In a previous study, a cohort of 83 patients with MTS were randomized to receive anterior temporal resection (36 patients) and medical management (43 patients). The results showed that 72% of patients who underwent surgery achieved favorable seizure-free outcomes compared to 23% in the non-surgical management group [85]. Consistent evidence was demonstrated by Wiebe et al. in a randomized controlled trial, which showed a 58% rate of seizure-free outcomes in the surgical group compared to 8% in the medical group [86]. The majority of seizure relapses occurred within the first year after surgery, with only a minority occurring after 1 year with causes such as withdrawal of anti-seizure medications [87].

While there are no significant differences in seizure control between ATL and SAH in the adult population [88], SAH has a worse prognosis of seizure control in children than in adults (33% vs. 71%), possibly due to the higher prevalence of dual pathology outside the hippocampus [89]. Conversely, ATL is effective in seizure control in 75% of children under the age of 12 [90].

In addition to surgical intervention, minimally invasive strategies have been used with lower morbidity and mortality rates.

Radiosurgery, which involves the application of radiation in an specific area of the brain, has proven effective, but offers delayed seizure control in about 9 to 24 months. Adverse effects described are transient increases in partial seizure and radiation-induced edema [91]. Stereo-electro-encephalography-guided radiofrequency thermocoagulation is a radiosurgery technique, which consists of thermocoagulate epileptogenic zones using radiofrecuency, with less and lower durable effects compared to ATL, although results vary among studies [92].

Deep brain stimulation (DBS) consists of targeting seizure onset zones and decreasing epileptiform activity by electrical stimulation [93]. This intervention exerts seizure control effects through activation of GABA receptors, thereby decreasing epileptogenesis, with less cognitive consequences [94, 95].

Magnetic resonance-guided laser interstitial thermal therapy (MR-gLiTT) is a promising therapeutic approach for refractory epilepsy and brain tumors. It is increasingly used as a new minimally invasive technique through colocation of an intracranial laser diode. However, MR-gLiTT does not appear to be more efficient than open surgery. More studies are needed to evaluate MR-gLiTT and other minimally invasive techniques for future applications [96].

### Complications of MTS surgery

Neurocognitive outcomes following surgery for MTS have been extensively studied, particularly in relation to the surgical procedure used. It is well-known that surgery for MTS can induce a risk of postoperative memory deficits, especially in the cases of dominant hemisphere resection, which can impact verbal memory [97].

Recent data have shown heterogeneity in the relationship between surgical procedure and neurocognitive outcomes. Mathon et al. found that the risks of cognitive and verbal memory impairments are greater in patients receiving ATL compared to those receiving SAH [98]. However, a retrospective study of neurocognitive outcomes in MTLE patients did not find a significant difference between the two surgical techniques in terms of intelligence quotient, verbal and visuospatial memory, and naming. There is a trend toward better preservation of naming ability in patients receiving SAH on the dominant side [99]. As a result, there is no consensus on the neurocognitive outcomes of SAH and ATL, and further research is needed to better understand the potential interactions between surgical approach and long-term neuropsychological outcomes [100].

Other variables have also been studied for their associations with memory outcomes. Even mild contralateral hippocampal abnormalities have been associated with postoperative verbal memory problems. Higher rates of postoperative seizures have been linked to verbal and visual memory impairment. Shorter epilepsy duration, younger age, and withdrawal of anti-seizure medications are predictors of better memory outcomes [101–103].

Visual field defects (VFD) following ATL have been described, with the most common deficit being contralateral superior homonymous quadrantanopia. The incidence of clinically symptomatic VFD has been reported to be as low as 8%. Yam et al. studied a cohort of patients who had received ATL and found that 35% of them had severe VFD > 90 degrees postoperatively. However, approximately 38% of these patients experienced improvement, with an average improvement of 38 degrees within the first year [104–106].

## Conclusions

MTS is a significant cause of epilepsy, and it often presents as a refractory form of epilepsy that does not respond well to standard anti-serizure medications. Due to the clinical burdens and its impact on quality of life, early diagnosis and management are crucial. A multidisciplinary team involving epileptologists, neurologists, neurosurgeons, neuropsychologists, and other healthcare professionals is needed to provide comprehensive care and make individualized treatment plans for individuals with MTS. The multidisciplinary efforts include a combination of medication management, lifestyle modifications, surgical interventions, and supportive therapies, to optimize seizure control and improve the overall outcomes of patients.

## Data Availability

Not applicable.

## Abbreviations

ATL: Anterior temporal lobectomy
DBS: Deep brain stimulation
EEG: Electroencephalogram
FDG-PET: 18F-fluorodeoxyglucose positron emission tomography
FLAIR: Fluid-attenuated inversion recovery
HS: Hippocampal sclerosis
IPL: Initial precipitating lesions
MRS: Proton-magnetic resonance spectroscopy
MRI: Magnetic resonance imaging
MR-gLiTT: Magnetic resonance-guided laser interstitial thermal therapy
MTS: Mesial temporal sclerosis
MTLE: Mesial temporal lobe epilepsy
PMRS: Proton-magnetic resonance spectroscopy
SAH: Selective amygdalohippocampectomy
TLE: Temporal lobe epilepsy
VFD: Visual field defects

## References

[1] Sztriha L, Gururaj AK, Bener A, Nork M. Temporal lobe epilepsy in children: etiology in a cohort with new-onset seizures. Epilepsia. 2002;43(1):75–80. 11879390 10.1046/j.1528-1157.2002.24201.x

[2] Falconer MA, Serefetinides EA, Corsellis JA. Etiology and pathogenesis of temporal lobe epilepsy. Arch Neurol. 1964;19:2333–2248. 10.1001/archneur.1964.00460150003001 14106980

[3] Bratz E. Ammonshornbefunde bei epileptischen. Archiv f Psychiatrie. 1899;31:820–36.

[4] Bouchet C, Cazavieilh J. De L’epilpsie consideree dans ses raports avec l’alienation mentale. Recherche sur la nature et le siege de ces deux maladies. Arch Gen Med. 1825;9:510–42.

[5] Sommer W. Erkrankung des ammonshorns als aetiologisches moment der epilepsie. Arch Psychiatrist Nervenkr. 1880;10:631–75.

[6] Stauder KH. Epilepsie und schlafenlappen. Arch Psychiat Nervenkrankh. 1935;104:181–211.

[7] Aaberg KM, Gunnes N, Bakken IJ, Lund Søraas C, Berntsen A, Magnus P, et al. Incidence and prevalence of childhood epilepsy: a nationwide cohort study. Pediatrics. 2017;139(5):e20163908. 28557750 10.1542/peds.2016-3908

[8] Nickels KC, Wong-Kisiel LC, Moseley BD, Wirrell EC. Temporal lobe epilepsy in children. Epilepsy Res Treat. 2012;2012:16. 10.1155/2012/849540 PMC3420576 22957247

[9] Temporal lobe epilepsy. Epilepsy Foundation. 2023. https://www.epilepsy.com/what-is-epilepsy/syndromes/temporal-lobe-epilepsy. Accessed 24 May 2023.

[10] Lee Y-J, Lee JS. Temporal lobe epilepsy surgery in children versus adults: from etiologies to outcomes. Korean J Pediatr. 2013;56(7):275–81. 23908666 10.3345/kjp.2013.56.7.275 PMC3728445

[11] González De La Aleja Tejera J, Sepúlveda Sánchez JM, Simón De Las Heras R, Muñoz González A, Saiz Díaz R. A, Rodríguez Peña-Marín M, et al. Epilepsia del lóbulo temporal. Clasificación etiológica en 61 pacientes en edad pediátrica. An Pediatría. 2008;69(3):227-31. 10.1157/13125816 18775267

[12] Simon Harvey A, Berkovic SF, Wrennall JA, Hopkins LJ. Temporal lobe epilepsy in childhood: clinical, EEG, and neuroimaging findings and syndrome classification in a cohort with new-onset seizures. Neurology. 1997;49(4):960–8. 9339674 10.1212/wnl.49.4.960

[13] Kwan P, Brodie MJ. Early identification of refractory epilepsy. N Engl J Med. 2000;342(5):314–9. 10660394 10.1056/NEJM200002033420503

[14] López-Rivera JA, Smuk V, Leu C, Nasr G, Vegh D, Stefanski A, et al. Incidence and prevalence of major epilepsy-associated brain lesions. Epilepsy Behav Rep. 2022;18:100527. 35243289 10.1016/j.ebr.2022.100527 PMC8885987

[15] Ben-Ari Y, Tremblay E, Ottersen OP, Meldrum BS. The role of epileptic activity in hippocampal and remote cerebral lesions induced by kainic acid. Brain Res. 1980;191(1):79–97. 7378761 10.1016/0006-8993(80)90316-9

[16] Golarai G, Cavazos JE, Sutula TP. Activation of the dentate gyrus by pentylenetetrazol evoked seizures induces mossy fiber synaptic reorganization. Brain Res. 1992;593(2):257–64. 1450933 10.1016/0006-8993(92)91316-7

[17] Molnár P, Nadler JV. Mossy fiber-granule cell synapses in the normal and epileptic rat dentate gyrus studied with minimal laser photostimulation. J Neurophysiol. 1999;82(4):1883–94. 10515977 10.1152/jn.1999.82.4.1883

[18] Cavazos JE, Sutula TP. Progressive neuronal loss induced by kindling: a possible mechanism for mossy fiber synaptic reorganization and hippocampal sclerosis. Brain Res. 1990;527(1):1–6. 2282474 10.1016/0006-8993(90)91054-k

[19] Olney JW. Excitotoxins, an overview. In: Fuxe K, Roberts P, SchwarcL R, editors. Excitotoxins. Wenner-gren center international symposium series. London: Palgrave Macmillan; 1984. p. 82–96.

[20] Sloviter RS. Decreased hippocampal inhibition and a selective loss of interneurons in experimental epilepsy. Science. 1987;235(4784):73–6. 2879352 10.1126/science.2879352

[21] Scharfman HE. Electrophysiological evidence that dentate hilar mossy cells are excitatory and innervate both granule cells and interneurons. J Neurophysiol. 1995;74(1):179–94. 7472322 10.1152/jn.1995.74.1.179

[22] Williamson A, Spencer DD, Shepherd GM. Comparison between the membrane and synaptic properties of human and rodent dentate granule cells. Brain Res. 1993;622(1):194–202. 8242356 10.1016/0006-8993(93)90819-9

[23] Liu Z, Mikati M, Holmes GL. Mesial temporal sclerosis: pathogenesis and significance. Pediatr Neurol. 1995;12(1):5–16. 7748361 10.1016/0887-8994(94)00122-i

[24] Sloviter RS. The functional organization of the hippocampal dentate gyrus and its relevance to the pathogenesis of temporal lobe epilepsy. Ann Neurol. 1994;35(6):640–54. 8210220 10.1002/ana.410350604

[25] Babb TL, Brown WJ. Pathological findings in epilepsy. In: Engel J Jr, editor. Surgical treatment of the epilepsies. New York: Raven; 1987. p. 511–40.

[26] Margerison JH, Corsellis JA. Epilepsy and the temporal lobes. A clinical, electroencephalographic and neuropathological study of the brain in epilepsy, with particular reference to the temporal lobes. Brain. 1966;89(3):499–530. 5922048 10.1093/brain/89.3.499

[27] Cendes F, Cook MJ, Watson C, Andermann F, Fish DR, Shorvon SD, et al. Frequency and characteristics of dual pathology in patients with lesional epilepsy. Neurology. 1995;45:2058–64. 7501159 10.1212/wnl.45.11.2058

[28] Li LM, Cendes F, Andermann F, Watson C, Fish DR, Cook MJ, et al. Surgical outcome in patients with epilepsy and dual pathology. Brain. 1999;122(Pt 5):799–805. 10355666 10.1093/brain/122.5.799

[29] Mohamed A, Wyllie E, Ruggieri P, Kotagal P, Babb T, Hilbig A, et al. Temporal lobe epilepsy due to hippocampal sclerosis in pediatric candidates for epilepsy surgery. Neurology. 2001;56(12):1643–9. 11425928 10.1212/wnl.56.12.1643

[30] Lewis DV, Shinnar S, Hesdorffer DC, Bagiella E, Bello JA, Chan S, et al. Hippocampal sclerosis after febrile status epilepticus: the FEBSTAT study. Ann Neurol. 2014;75(2):178–85. 24318290 10.1002/ana.24081 PMC3980500

[31] Aulická S, Česká K, Šána J, Siegl F, Brichtová E, Ošlejšková H, et al. Cytokine-chemokine profiles in the hippocampus of patients with mesial temporal lobe epilepsy and hippocampal sclerosis. Epilepsy Res. 2022;180:106858. 35026708 10.1016/j.eplepsyres.2022.106858

[32] Cavanagh JB, Meyer A. Aetiological aspects of Ammon’s horn sclerosis associated with temporal lobe epilepsy. Br Med J. 1956;2(5006):1403–7. 13374345 10.1136/bmj.2.5006.1403 PMC2035906

[33] Kotsenas AL, Watson RE, Pittock SJ, Britton JW, Hoye SL, Quek AM, et al. MRI findings in autoimmune voltage-gated potassium channel complex encephalitis with seizures: one potential etiology for mesial temporal sclerosis. AJNR Am J Neuroradiol. 2014;35(1):84–9. 23868165 10.3174/ajnr.A3633 PMC7966496

[34] Mathern GW, Pretorius JK, Babb TL. Influence of the type of initial precipitating injury and at what age it occurs on course and outcome in patients with temporal lobe seizures. J Neurosurg. 1995;82(2):220–7. 7815149 10.3171/jns.1995.82.2.0220

[35] Blümcke I, Pauli E, Clusmann H, Schramm J, Becker A, Elger C, et al. A new clinico-pathological classification system for mesial temporal sclerosis. Acta Neuropathol. 2007;113(3):235–44. 17221203 10.1007/s00401-006-0187-0 PMC1794628

[36] Davies KG, Hermann BP, Dohan FC Jr, Foley KT, Bush AJ, Wyler AR. Relationship of hippocampal sclerosis to duration and age of onset of epilepsy, and childhood febrile seizures in temporal lobectomy patients. Epilepsy Res. 1996;24(2):119–26. 8796360 10.1016/0920-1211(96)00008-3

[37] Sagar HJ, Oxbury JM. Hippocampal neuron loss in temporal lobe epilepsy: correlation with early childhood convulsions. Ann Neurol. 1987;22(3):334–40. 3674798 10.1002/ana.410220309

[38] Kasperaviciute D, Catarino CB, Matarin M, Leu C, Novy J, Tostevin A, et al. Epilepsy, hippocampal sclerosis and febrile seizures linked by common genetic variation around SCN1A. Brain. 2013;136(Pt 10):3140–50. 24014518 10.1093/brain/awt233 PMC3784283

[39] Bruxel EM, Bruno DCF, do Canto AM, Geraldis JC, Godoi AB, Martin M, et al. Multi-omics in mesial temporal lobe epilepsy with hippocampal sclerosis: clues into the underlying mechanisms leading to disease. Seizure. 2021;90:34–50. 33722437 10.1016/j.seizure.2021.03.002

[40] Striano P, Nobile C. Whole-exome sequencing to disentangle the complex genetics of hippocampal sclerosis-temporal lobe epilepsy. Neurol Genet. 2018;4:241. 10.1212/NXG.0000000000000241 PMC5999347 29904719

[41] Yam KK, Leung WA, Zhu XL, Fung LE. Drug resistant epilepsy with mesial temporal sclerosis as possible late neurological complication in two AML survivors after stem cell transplantation. Epilepsy Behav Case Rep. 2018;10:71–7. 30073146 10.1016/j.ebcr.2018.05.001 PMC6068318

[42] de Lanerolle NC, Kim JH, Williamson A, Spencer SS, Zaveri HP, Eid T, et al. A retrospective analysis of hippocampal pathology in human temporal lobe epilepsy: evidence for distinctive patient subcategories. Epilepsia. 2003;44(5):677–87. 12752467 10.1046/j.1528-1157.2003.32701.x

[43] Blümcke I, Thom M, Aronica E, Armstrong DD, Bartolomei F, Bernasconi A, et al. International consensus classification of hippocampal sclerosis in temporal lobe epilepsy: a task force report from the ILAE Commission on diagnostic methods. Epilepsy. 2013;54(7):1315–29. 10.1111/epi.12220 23692496

[44] Wieser HG, Commission on Neurosurgery of Epilepsy. ILAE commission report. Mesial temporal lobe epilepsy with hippocampal sclerosis. Epilepsy. 2004;45:695–714. 10.1111/j.0013-9580.2004.09004.x 15144438

[45] Gibbs FA, Gibbs EL, Lennox WG. Epilepsy: a paroxysmal cerebral dysrhythmia. Brain. 1937;60:377–88. 10.1016/s1525-5050(02)00050-1 12609340

[46] Nayak CS, Bandyopadhyay S. Mesial temporal lobe epilepsy. In: StatPearls. Treasure Island (FL): StatPearls Publishing; 2022. https://www.ncbi.nlm.nih.gov/books/NBK554432/. Accessed 24 May 2023. 32119319

[47] Alsaadi TM, Marquez AV. Psychogenic nonepileptic seizures. Am Fam Physician. 2005;72(5):849–56. 16156345

[48] Berkovic SF, Mcintosh AM, Kalnins RM, Jackson GD, Fabinyi GCA, Brazenor GA, et al. Preoperative MRI predicts outcome of temporal lobectomy: an actuarial analysis. Neurology. 1995;45(7):1358–63. 7617198 10.1212/wnl.45.7.1358

[49] Kuzniecky R, Burgard S, Faught E, Morawetz R, Bartolucci A. Predictive value of magnetic resonance imaging in temporal lobe epilepsy surgery. Arch Neurol (Chicago). 1993;50(1):65–9. 8418802 10.1001/archneur.1993.00540010059018

[50] Malmgren K, Thom M. Hippocampal sclerosis-origins and imaging. Epilepsia (Copenhagen). 2012;53(s4):19–33. 10.1111/j.1528-1167.2012.03610.x 22946718

[51] Van Paesschen W. Qualitative and quantitative imaging of the hippocampus in mesial temporal lobe epilepsy with hippocampal sclerosis. Neuroimaging Clin N Am. 2004;14(3):373–400. 15324854 10.1016/j.nic.2004.04.004

[52] Briellmann RS, Kalnins RM, Berkovic SF, Jackson GD. Hippocampal pathology in refractory temporal lobe epilepsy: T2-weighted signal change reflects dentate gliosis. Neurology. 2002;58:265–71. 11805255 10.1212/wnl.58.2.265

[53] Hirai T, Korogi Y, Yoshizumi K, Shigematsu Y, Sugahara T, Takahashi M. Limbic lobe of the human brain: evaluation with turbo fluid-attenuated inversion-recovery MR imaging. Radiology. 2000;215:470–5. 10796927 10.1148/radiology.215.2.r00ma06470

[54] Meiners LC, Van Gils A, Jansen GH, De Kort G, Witkamp TD, Ramos LM, et al. Temporal lobe epilepsy: the various MR appearances of histologically proven mesial temporal sclerosis. AJNR Am J Neuroradiol. 1994;15:1547–55. 7985576 PMC8334415

[55] Chan S, Erickson JK, Yoon SS. Limbic system abnormalities associated with mesial temporal sclerosis: a model of chronic cerebral changes due to seizures. Radiographics. 1997;17(5):1095–110. 9308104 10.1148/radiographics.17.5.9308104

[56] Cendes F, Cascino GD. MRI signs of hippocampal sclerosis seen in healthy volunteers: what is the clinical relevance? Neurology. 2010;74(7):534–5. 20089942 10.1212/WNL.0b013e3181cff7b2

[57] Kobayashi E, Li LM, Lopes-Cendes I, Cendes F. Magnetic resonance imaging evidence of hippocampal sclerosis in asymptomatic, first-degree relatives of patients with familial mesial temporal lobe epilepsy. Arch Neurol. 2002;59:1891–4. 12470176 10.1001/archneur.59.12.1891

[58] Menzler K, Iwinska-Zelder J, Shiratori K, Jaeger RK, Oertel WH, HamerHM, et al. Evaluation of MRI criteria (1.5 T) for the diagnosis of hippocampal sclerosis in healthy subjects. Epilepsy Res. 2010;89:349–35. 10.1016/j.eplepsyres.2010.02.010 20307956

[59] Labate A, Gambardella A, Aguglia U, Condino F, Ventura P, Lanza P, et al. Temporal lobe abnormalities on brain MRI in healthy volunteers: a prospective case–control study. Neurology. 2010;74:553–7. 20089943 10.1212/WNL.0b013e3181cff747

[60] Granados Sánchez AM, Orejuela Zapata JF. Diagnosis of mesial temporal sclerosis: sensitivity, specificity and predictive values of the quantitative analysis of magnetic resonance imaging. Neuroradiol J. 2018;31(1):50–9. 28899220 10.1177/1971400917731301 PMC5789997

[61] Duncan JS, Winston GP, Koepp MJ, Ourselin S. Brain imaging in the assessment for epilepsy surgery. Lancet Neurol. 2016;15(4):420–33. 26925532 10.1016/S1474-4422(15)00383-X PMC6736670

[62] Farid N, Girard HM, Kemmotsu N, Smith ME, Magda SW, Lim WY, et al. Temporal lobe epilepsy: quantitative MR volumetry in detection of hippocampal atrophy. Radiology. 2012;264:542–50. 22723496 10.1148/radiol.12112638 PMC3401351

[63] Jack CR Jr, Sharbrough FW, Twomey CK, Cascino GD, Hirschorn KA, Marsh WR, et al. Temporal lobe seizures: lateralization with MR volume measurements of the hippocampal formation. Radiology. 1990;175:423–9. 2183282 10.1148/radiology.175.2.2183282

[64] Pardoe HR, Pell GS, Abbott DF, Jackson GD. Hippocampal volume assessment in temporal lobe epilepsy: how good is automated segmentation? Epilepsia. 2009;50:2586–92. 19682030 10.1111/j.1528-1167.2009.02243.x PMC3053147

[65] Kuzniecky RI, Bilir E, Gilliam F, Faught E, Palmer C, Morawetz R, et al. Multimodality MRI in mesial temporal sclerosis: relative sensitivity and specificity. Neurol. 1997;49(3):774–8. 10.1212/wnl.49.3.774 9305339

[66] Van Paesschen W, Revesz T, Duncan JS, King MD, Connelly A. Quantitative neuropathology and quantitative magnetic resonance imaging of the hippocampus in temporal lobe epilepsy. Ann Neurol. 1997;42:756–66. 9392575 10.1002/ana.410420512

[67] Xu MY, Ergene E, Zagardo M, Tracy PT, Wang H, Liu W, et al. Proton MR spectroscopy in patients with structural MRI-negative temporal lobe epilepsy. J Neuroimaging. 2015;25(6):1030–7. 26011809 10.1111/jon.12263

[68] Luther N, Rubens E, Sethi N, Kandula P, Labar DR, Harden C, et al. The value of intraoperative electrocorticography in surgical decision making for temporal lobe epilepsy with normal MRI. Epilepsia. 2011;52:941–8. 21480886 10.1111/j.1528-1167.2011.03061.x PMC3623284

[69] Mendes-Ribeiro JA, Soares R, Simões-Ribeiro F, Guimarães ML. Reduction in temporal N-acetylaspartate and creatine (or choline) ratio in temporal lobe epilepsy: does this 1H-magnetic resonance spectroscopy finding mean poor seizure control? J Neurol Neurosurg Psychiatry. 1998;65(4):518–22. 9771777 10.1136/jnnp.65.4.518 PMC2170285

[70] Fountas K, Tsougos I, Gotsis E, Giannakodimos S, Smith JR, Kapsalaki EZ. Temporal pole proton preoperative magnetic resonance spectroscopy in patients undergoing surgery for mesial temporal sclerosis. NeuroSurg Focus. 2012;32(3):E3. 22380857 10.3171/2012.1.FOCUS11327

[71] Willmann O, Wennberg R, May T, Woerman FG, Pohlmann-Eden B. The role of 1H magnetic resonance spectroscopy in pre-operative evaluation for epilepsy surgery a meta-analysis. Epilepsy Res. 2006;71(2–3):149–58. 16890408 10.1016/j.eplepsyres.2006.06.004

[72] Engel J Jr, Henry TR, Risinger MW, Mazziotta JC, Sutherling WW, Levesque MF, et al. Presurgical evaluation for partial epilepsy: relative contributions of chronic depth-electrode recording sversus FDG-PET and scalp-sphenoidal ictal. EEG Neurol. 1990;40:1670–7. 10.1212/wnl.40.11.1670 2122275

[73] Henry TR, Mazziotta JC, Engel J Jr. Interictal metabolic anatomy of mesial temporal lobe epilepsy. Arch Neurol. 1993;50:582–9. 8503794 10.1001/archneur.1993.00540060022011

[74] Achten E, Santens P, Boon P, De Coo D, Van De Kerckhove T, De Reuck J, et al. Single-voxel proton MR spectroscopy and positron emission tomography for lateralization of refractory temporal lobe epilepsy. AJNR Am J Neuroradiol. 1998;19:1–8. 9432150 PMC8337324

[75] Park SW, Chang KH, Kim HD, Song IC, Lee DS, Lee SK, et al. Lateralizing ability of single-voxel proton MR spectroscopy in hippocampal sclerosis: comparison with MR imaging and positron emission tomography. AJNR Am J Neuroradiol. 2001;22:625–31. 11290468 PMC7976017

[76] Willmann O, Wennberg R, May T, Woermann FG, Pohlmann-Eden B. The contribution of 18F-FDG PET in preoperative epilepsy surgery evaluation for patients with temporal lobe epilepsy a meta-analysis. Seizure. 2007;16:509–20. 17532231 10.1016/j.seizure.2007.04.001

[77] Kasasbeh A, Hwang EC, Steger-May K, Bandt SK, Oberhelman A, Limbrick D, et al. Association of magnetic resonance imaging identification of mesial temporal sclerosis with pathological diagnosis and surgical outcomes in children following epilepsy surgery. J Neurosurg Pediatr. 2012;9(5):552–61. 22546035 10.3171/2012.1.PEDS11447

[78] Tasch E, Cendes F, Li LM, Dubeau F, Andermann F, Arnold DL. Neuroimaging evidence of progressive neuronal loss and dysfunction in temporal lobe epilepsy. Ann Neurol. 1999;45:568–76. 10319878 10.1002/1531-8249(199905)45:5<568::aid-ana4>3.0.co;2-p

[79] Cross JH, Connelly A, Jackson GD, Johnson CL, Neville BGR, Gadian DG. Proton magnetic resonance spectroscopy in children with temporal lobe epilepsy. Ann Neurol. 1996;39(1):107–13. 8572655 10.1002/ana.410390116

[80] Asadi-Pooya AA, Stewart GR, Abrams DJ, Sharan A. Prevalence and incidence of drug-resistant mesial temporal lobe Epilepsy in the United States. World Neurosurg. 2017;99:662–6. 28034810 10.1016/j.wneu.2016.12.074

[81] Stephen LJ, Kwan P, Brodie MJ. Does the cause of localisation-related epilepsy influence the response to antiepileptic drug treatment? Epilepsia. 2001;42(3):357–62. 11442153 10.1046/j.1528-1157.2001.29000.x

[82] Wiebe S, Blume WT, Girvin JP, Eliasziw M. Effectiveness and efficiency of surgery for temporal lobe epilepsy study group. A randomized, controlled trial of surgery for temporal-lobe epilepsy. N Engl J Med. 2001;345(5):311–8. 11484687 10.1056/NEJM200108023450501

[83] Mathon B, Bédos Ulvin L, Adam C, Baulac M, Dupont S, Navarro V, et al. Surgical treatment for mesial temporal lobe epilepsy associated with hippocampal sclerosis. Rev Neurol (Paris). 2015;171(3):315–25. 25746582 10.1016/j.neurol.2015.01.561

[84] Yasargil MG, Teddy PJ, Roth P. Selective amygdalohippocampectomy. Operative anatomy and surgical technique. Adv Tech Stand Neurosurg. 1985;12:93–123. 4084377 10.1007/978-3-7091-7008-3_2

[85] Kumlien E, Doss RC, Gates JR. Treatment outcome in patients with mesial temporal sclerosis. Seizure. 2002;11(7):413–7. 12237065 10.1053/seiz.2001.0614

[86] Wiebe S, Blume WT, Girvin JP, Eliasziw M. A randomized, controlled trial of surgery for temporal lobe epilepsy. N Engl J Med. 2001;345:311–8. 11484687 10.1056/NEJM200108023450501

[87] Jutila L, Immonen A, Mervaala E, Partanen J, Partanen K, Puranen M, et al. Long term outcome of temporal lobe epilepsy surgery: analyses of 140 consecutive patients. J Neurol Neurosurg Psychiatry. 2002;73(5):486–94. 12397139 10.1136/jnnp.73.5.486 PMC1738104

[88] Wieser HG, Ortega M, Friedman A, Yonekawa Y. Long-term seizure outcomes following amygdalohippocampectomy. J Neurosurg. 2003;98:751–63. 12691400 10.3171/jns.2003.98.4.0751

[89] Datta A, Sinclair DB, Wheatley M, Jurasek L, Snyder T, Quigley D, et al. Selective amygdalohippocampectomy: Surgical outcome in children versus adults. Can J Neurol Sci. 2009;36(2):187–91. 19378712

[90] Duchowny M, Levin B, Jayakar P, Resnick T, Alvarez L, Morrison G, et al. Temporal lobectomy in early childhood. Epilepsia. 1992;33:298–303. 1547759 10.1111/j.1528-1157.1992.tb02319.x

[91] Gianaris T, Witt T, Barbaro NM. Radiosurgery for medial temporal lobe Epilepsy resulting from mesial temporal sclerosis. Neurosurg Clin N Am. 2016;27(1):79–82. 26615110 10.1016/j.nec.2015.08.011

[92] Fan X, Shan Y, Lu C, An Y, Wang Y, Du J, et al. Optimized SEEG-guided radiofrequency thermocoagulation for mesial temporal lobe epilepsy with hippocampal sclerosis. Seizure. 2019;71:304–11. 31521052 10.1016/j.seizure.2019.08.011

[93] Foutz TJ, Wong M. Brain stimulation treatments in epilepsy: Basic mechanisms and clinical advances. Biomed J. 2022;45(1):27–37. 34482013 10.1016/j.bj.2021.08.010 PMC9133258

[94] Wang YH, Chen SC, Wei PH, Yang K, Fan XT, Meng F, et al. Stereotactic EEG-guided radiofrequency thermocoagulation versus anterior temporal lobectomy for mesial temporal lobe epilepsy with hippocampal sclerosis: study protocol for a randomised controlled trial. Trials. 2021;22(1):425. 34187524 10.1186/s13063-021-05378-3 PMC8244214

[95] Velasco F, Saucedo-Alvarado PE, Vazquez-Barron D, Trejo D, Velasco AL. Deep brain stimulation for refractory temporal lobe epilepsy current status and future trends. Front Neurol. 2022;13:796846. 35280275 10.3389/fneur.2022.796846 PMC8904383

[96] Consales A, Cognolato E, Pacetti M, Mancardi MM, Tortora D, Di Perna G, et al. Magnetic resonance-guided laser interstitial thermal therapy (MR-gLiTT) in pediatric epilepsy surgery: state of the art and presentation of Giannina Gaslini Children’s Hospital (Genoa, Italy) series. Front Neurol. 2021;12:739034. 34764929 10.3389/fneur.2021.739034 PMC8577648

[97] Helmstaedter C, Richter S, Röske S, Oltmanns F, Schramm J, Lehmann T. Differential effects of temporal pole resection with amygdalohippocampectomy versus selective amygdalohippocampectomy on material-specific memory in patients with mesial temporal lobe epilepsy. Epilepsia (Copenhagen). 2008;49(1):88–97. 10.1111/j.1528-1167.2007.01386.x 17941848

[98] Mathon B, Bielle F, Samson S, Plaisant O, Dupont S, Bertrand A, et al. Predictive factors of long-term outcomes of surgery for mesial temporal lobe epilepsy associated with hippocampal sclerosis. Epilepsia (Copenhagen). 2017;58(8):1473–85. 10.1111/epi.13831 28656696

[99] Mansouri A, Fallah A, McAndrews MP, Cohn M, Mayor D, Andrade D, et al. Neurocognitive and seizure outcomes of selective amygdalohippocampectomy versus anterior temporal lobectomy for mesial temporal lobe epilepsy. Epilepsy Res Treat. 2014;2014:306382–8. 25349728 10.1155/2014/306382 PMC4198822

[100] Alexandratou I, Patrikelis P, Messinis L, Alexoudi A, Verentzioti A, Stefanatou M, et al. Long-term neuropsychological outcomes following temporal lobe Epilepsy surgery: an update of the literature. Healthc (Basel). 2021;9(9):1156. 10.3390/healthcare9091156 PMC8466433 34574930

[101] Incisa della Rocchetta A, Gadian DG, Connelly A, Polkey CE, Jackson GD, Watkins KE, et al. Verbal memory impairment after right temporal lobe surgery: role of contralateral damage as revealed by 1H magnetic resonance spectroscopy and T2 relaxometry. Neurology. 1995;45:797–802. 7723973 10.1212/wnl.45.4.797

[102] Baxendale S, Thompson PJ, Duncan JS. Neuropsychological function in patients who have had epilepsy surgery: a long-term follow-up. Epilepsy Behav. 2012;23:24–9. 22100066 10.1016/j.yebeh.2011.10.021

[103] Salvato G, Scarpa P, Francione S, Mai R, Tassi L, Scarano E, et al. Declarative long-term memory and the mesial temporal lobe: insights from a 5-year postsurgery follow-up study on refractory temporal lobe epilepsy. Epilepsy Behav. 2016;64:102–9. 27736656 10.1016/j.yebeh.2016.08.029

[104] Falconer MA, Wilson JL. Visual field changes following anterior temporal lobectomy: their significance in relation to Meyer’s loop of the optic radiation. Brain. 1958;81:1–14. 13523001 10.1093/brain/81.1.1

[105] Tecoma ES, Laxer KD, Barbaro NM, Plant GT. Frequency and characteristics of visual field deficits after surgery for mesial temporal sclerosis. Neurology. 1993;43:1235–8. 8170572 10.1212/wnl.43.6.1235

[106] Yam D, Nicolle D, Steven DA, Lee D, Hess T, Burneo JG. Visual field deficits following anterior temporal lobectomy: long-term follow‐up and prognostic implications. Epilepsia (Copenhagen). 2010;51(6):1018–23. 10.1111/j.1528-1167.2009.02427.x 20074232

